# Transdifferentiation of human adipose-derived mesenchymal stem cells into oligodendrocyte progenitor cells

**Published:** 2018-01-05

**Authors:** Nazem Ghasemi

**Affiliations:** Department of Anatomical Sciences, School of Medicine, Isfahan University of Medical Sciences, Isfahan, Iran

**Keywords:** Adult Stem Cells, Cell Differentiation, Oligodendroglia

## Abstract

**Background:** Stem cell-based therapy is a new method for the treatment of neurodegenerative diseases such as multiple sclerosis (MS). Human adipose-derived stem cells (hADSCs) are a kind of adult stem cells which have a higher frequency in the fat tissue and have the ability to differentiate into other cell types outside their lineage. Due to some serious adverse events of cell-based therapy such as tumorigenic potential, the aim of this study was to evaluate of hADSCs differentiation into oligodendrocytes as a valuable way for future cell transplantation.

**Methods:** hADSC were isolated from lipoaspirate samples of human abdominal fat. After hADSC characterization via flow cytometry, the cells were induced to oligodendrocytes using a special differentiation medium. Finally, 3-(4,5-dimethylthiazol-2-yl)-2,5-diphenyltetrazolium bromide (MTT), immunocytochemistry, and real-time polymerase chain reaction (RT-PCR) techniques were used for the evaluation of differentiated cells.

**Results:** Flow cytometry indicated that hADSCs were CD105- and CD49-positive, but were negative for CD31 and CD45 markers. In addition, immunocytochemistry analysis revealed that a high percent of differentiated cells expressed oligodendrocyte progenitor cells markers [A2B5 and oligodendrocyte transcription factor (Olig2)] which were significantly higher than myelin basic protein (MBP) which is mature oligodendrocytes marker. Moreover, a very low percentage of differentiated cells expressed glial fibrillary acidic protein (GFAP) marker. Finally, real-time reverse transcription PCR analysis confirmed the results of immunocytochemistry.

**Conclusion:** Since hADSCs have the potential to differentiate into multi-lineage cells and due to their additional characteristics such as immunomodulatory and neuroprotective properties, it seems that these cells may be an ideal cell source for oligodendrocytes differentiation.

## Introduction

Cell-based treatment is a novel idea for the treatment of demyelinating diseases such as multiple sclerosis (MS). The conclusive purpose of this strategy is the cell substitution and upregulation of neurotropic factors as well as downregulation of apoptotic agents. MS is one of the most common autoimmune dysmyelinating disorders in central nervous system (CNS) which affects men more than women (sex ratio of 2.5:1).^1^^,^^2^ The use of disease-modifying drugs cannot prevent MS progression.^3^^,^^4^ So, stem cell-base therapy is proposed to provide a cure for MS.^5^^-^^7^

In recent studies, human embryonic stem cells, human bone-marrow-derived mesenchymal stem cells (BM-MSCs), and human placental mesenchymal stem cells were transplanted in animal model of MS.^8^^,^^9^ Despite the efficiency of stem cell transplantation, serious side effects which may occur following the stem cell transplantation (such as tumorigenesis) cannot be denied.^10^ Therefore, the use of fully differentiated cells instead of stem cells is a necessity. Laboratory studies proven that human Wharton’s jelly stem cells (hWJ-SCs) and human dental pulp stem cells (DPSCs) are able to differentiate into other cells outside of their lineage such as oligodendrocyte progenitor cells.^11^^,^^12^ In addition, these cells, when transplanted in animal model of MS, are able to promote the regeneration of myelin sheaths, and significantly reduce the clinical signs of MS.

Since the frequency of hWJ-SCs-, DPSCs-, and BM-MSCs in-related tissue is low, and the isolation of these cells is a difficult process, many researchers have paid special attention to human adipose-derived stem cells (hADSCs). 

hADSCs are specie of adult stem cells which have particular features including immunomodulatory and neuroprotective effects. In addition, these cells are able to differentiate into other cells in the body as well as a potency to generate many identified neurotrophic agents such as brain-derived neurotrophic factor (BDNF), nerve growth factor (NGF), and glial cell line-derived neurotrophic factor (GDNF).^5^^,^^13^ Meanwhile, some studies demonstrated that hADSCs are able to cross the blood-brain barrier and exert their action.^5^^,^^6^

Collectively, available data suggest that if hADSCs have the ability to differentiate into oligodendrocyte cells, these cells can be used as an ideal source of stem cells for MS treatment. Thus, the aim of this in-vitro study was to evaluate hADSCs differentiation into oligodendrocyte cells in order to access a valuable cell source for cell-based treatment in MS disease.

## Materials and Methods

***Stem cell isolation and culture:*** All procedures used in this study were approved by the Ethics Committee of Isfahan University of Medical Sciences, Isfahan, Iran (ethics code: 194267). After getting informed consent from healthy female donors (age range of 20-40 years) who referred to Alzahra hospital (Isfahan) for cesarean surgery, hADSCs were harvested from abdominal fat, and cultured according to our previous study.^5^ Briefly, after washing with phosphate-buffered saline (PBS) (Sigma-Aldrich, UK), the samples were treated with 0.075% collagenase type I (Sigma-Aldrich, UK) for enzymatic degradation. In the following, the enzyme activity was neutralized with Dulbecco’s Modified Eagles Medium (DMEM/F12) (Gibco, UK) contained 10% fetal bovine serum (FBS) (Gibco, UK), and then centrifuged for 10 minutes. Finally, the cell pellet was resuspended in DMEM/F12, 10% FBS, and 1% penicillin/streptomycin solution, and was cultured under standard conditions.

***Flow cytometry and cell characterization : ***For this purpose, 1 × 10^6^ hADSCs (within 3 passages) were fixed in 2% paraformaldehyde (Sigma-Aldrich, UK) for 25 minutes, and after washing with PBS, the samples were incubated with respective fluorochrome-conjugated antibodies against CD105, CD49, CD31, and CD45 (Chemicon, Temecula, CA, USA) for 30 minutes. In addition, nonspecific fluorescein isothiocyanate (FITC)-conjugated immunoglobulin G (IgG) was used for isotype control. After incubation, the cells were washed and resuspended in PBS. Finally, the percentage of fluorescent cells was analyzed using a flow cytometer (Becton Dickinson, San Jose, CA, USA).

***Induction of hADSCs into oligodendrocyte progenitor cells:*** According to our previous protocol,^14^^,^^15^ 1 × 10^4^ hADSCs/cm^2^ in the fifth passage were seeded into cell culture special plates, and cultured in present of DMEM/F12 which supplemented with 10 µl/ml N_2_ (Gibco, UK), 10 ng/ml human recombinant epidermal growth factor (EGF) (Biolegend, UK), and penicillin/streptomycin (SPN Solutions, Tysons Corner, VA, USA) in standard incubator for 3 days. After this time, trypsin-ethylenediaminetetraacetic acid (EDTA) solution (0.25%-0.02%) was used to detach the cells from the wells. Then, the cells were plated in plastic dish at a density of 2 × 10^2^ cells/cm^2^ in presence of neurobasal medium (Life Technologies, UK) containing 20 ng/ml basic fibroblast growth factor (bFGF) (Pepro Tech, UK), B27 2% (Gibco, UK), 20 ng/ml EGF (Pepro Tech, UK), 10 U/ml of penicillin, and 10 mg/ml streptomycin for 18 days. Finally, the cells in previous stage were cultured in 12 well tissue culture plates which coated with poly-L-Lysine (Sigma-Aldrich, UK) in a differentiation medium consisting of DMEM/F12, 1 × non-essential amino acids (NEAA) (Gibco, UK), L-glutamine (2 mM) (Gibco, UK), 1 × N_2_ (Invitrogen, Carlsbad, CA, USA), 1 × B27 (Gibco, UK), sonic hedgehog (SHH: 200 ng/ml) (Sigma-Aldrich, UK), retinoic acid (2 µM) (Sigma-Aldrich, UK), in standard condition for 10 days and in second medium with DMEM/F12, 1 × NEAA, L-glutamine (2 mM), 1 × N_2_, 1 × B27, neurotrophin-3 (NT3) (30 ng/ml) (Biolegend, UK), and platelet-derived growth factor alpha (PDGFα) (10 ng/ml) (Biolegend, UK) for 2 weeks. 

***3-(4,5-dimethylthiazol-2-yl)-2,5-diphenyltetrazolium bromide (MTT) assay:*** MTT assay was used for detection of cell viability before and after the final stage of cell differentiation. To this purpose, MTT solution (5 mg/ml) (Sigma-Aldrich, UK) was added to the hADSCs culture medium (control group) and to the differentiation medium (experimental group) at a dilution of 1:10 at 37 °C for 4 hours. Finally, the medium was replaced with 200 µl of dimethyl sulfoxide (DMSO) (Sigma-Aldrich, UK), and the absorbance of the solution in each well was detected using a microplate reader (Hiperion MPR 4+, Germany) at 540 nm.

***Immunocytochemistry analysis for oligodendrocyte characterization:*** To evaluate differentiation of hADSCs into oligodendrocytes, the differentiated cells ﬁxed first in 4% paraformaldehyde, and then cell permeabilized with 1% BSA/10% normal goat serum/0.3M glycine in 0.1% PBS-Tween. Then, the samples incubated with primary antibodies in humidified condition at 4 °C overnight. For this purpose, anti-A2B5 FITC antibody (1 µg/ml Abcam, Cambridge, MA, USA), anti-oligodendrocyte transcription factor (Olig2) FITC antibody (1:1000; Abcam, Cambridge, MA, USA), anti- myelin basic protein (MBP) FITC antibody (1:1000; Abcam, Cambridge, MA, USA), and anti-glial fibrillary acidic protein (GFAP) FITC antibody (1:1200; Abcam, Cambridge, MA, USA) were used. After cell washing with PBS, the samples were treated with goat anti-mouse FITC (1:500; Abcam, Cambridge, MA, USA)-conjugated secondary antibodies at room temperature for 1 hour and for 5 minutes with 4',6-diamidino-2-phenylindole (DAPI) in order to cell counting. Finally, the number of A2B5-, Olig2-, MBP-, and GFAP-positive cells were counted for a minimum of 200 cells per slide using fluorescence microscope (Olympus, BX51, Japan). Meanwhile, all immunocytochemistry studies were repeated at least twice, and undifferentiated hADSCs were used as the control sample.

***Real-time polymerase chain reaction (RT-PCR):*** At the end of differentiation procedure, the differentiated cells were subjected to real-time (SYBR Green) PCR. Briefly, total RNA was extracted from 1 × 10^5^ differentiated cells and 1 × 10^5^ undifferentiated cells using RNeasy micro Kit (Qiagen, Germany), and then cDNA synthesis was done with  Revert Aid First Strand cDNA Synthesis Kit (Fermentas, Burlington, ON, Canada). Specific genes, including Olig2, platelet-derived growth factor receptor alpha (PDGFRα), MBP, and astrocyte specific marker (GFAP), and a housekeeping gene glyceraldehyde-3-phosphate dehydrogenase (GAPDH) relative expression analysis was done by Thermal Cycler Rotor-Gene. Meanwhile, real-time specific primer pairs which used in this study were shown in table 1. 

**Table 1:** List of primers which used for real-time polymerase chain reaction (RT-PCR) analysis

**Gene primers**	**Sequence**
MBP	F: 5'-GTAGTAAGCCACTCCTTGACTG-3' R: 5'-GCAGAGAGGACTGTTGACAT-3'
Olig2	F: 5'-CGCAGCGAGCACCTCAAATCTAA-3' R: 5'-CCCAGGGATGATCTAAGCTCTCGAA-3'
PDGFRα	F: 5'- GTG GGA CAT TCA TTG CGG A-3' Rev: 5' AAG CTG GCA GAG GAT TAG G-3'
GFAP	F: 5'- CCGACAGCAGGTCCATGTG-3' Rev: 5'-GTTGCTGGACGCCATTGC-3'
GAPDH	F: 5'- GAAATCCCATCACCATCTTCCAGG-3' Rev: 5'-GAGCCCCAGCCTTCTCCATG-3'

MBP: Myelin basic protein; Olig2: Oligodendrocyte transcription factor; PDGFRα: Platelet-derived growth factor receptor α; GFAP: Glial fibrillary acidic protein; GAPDH: Glyceraldehyde-3-phosphate dehydrogenase

Data analysis was performed via SPSS software (version 20, IBM Corporation, Armonk, NY) using independent samples t test and one-way analysis of variance (ANOVA). All data were presented as mean ± standard error of the mean (SEM), and values of P ≤ 0.050 was considered as statistically significant.

## Results

**Figure 1:**
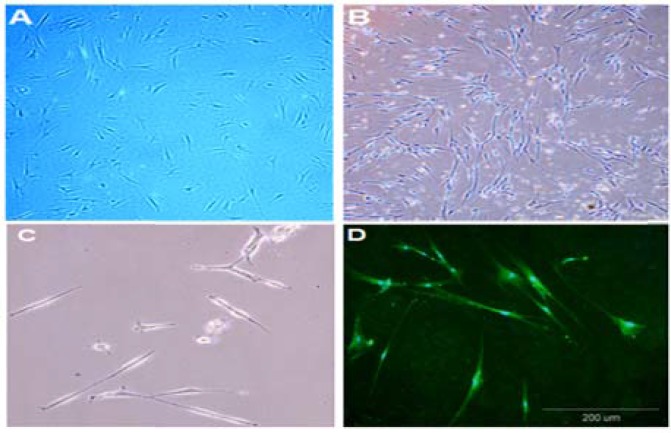
Morphological changes of human adipose-derived stem cells (hADSCs) into oligodendrocyte precursor cells

***Cell characterization: ***As shown in figure 1-A, before induction of hADSCs differentiation, these cells exhibited fibroblast-like morphology. Moreover, morphological changes were observed in the all stage of cell differentiation. In primary stages of differentiation, cellular networks and in end stage of differentiation, multipolar cells were seen (Figure 1-B, C, and D). 

The cultured hADSCs in passage three (A), the differentiated cells in early stage of oligodendrocyte differentiation (B), and in the end of differentiation process (C and D) are seen. Images were taken at ×40 magnification in A and B, and ×100 magnification in C and D (Scale bar: 200 µm).

**Figure 2:**
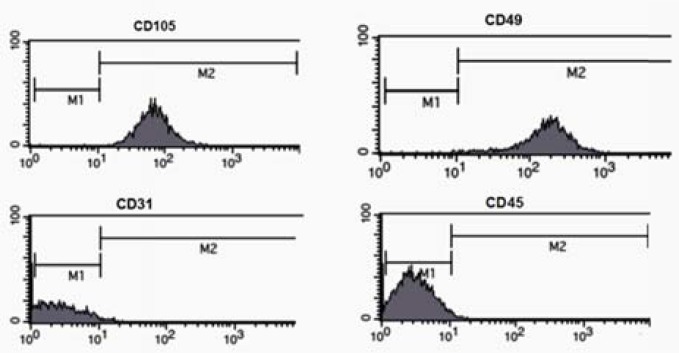
Flow cytometric analysis of human adipose-derived stem cells (hADSCs) which were CD105- and CD49-positive but were negative for CD31 and CD45.

In addition, flow cytometry showed that hADSCs were CD105- and CD49-positive, but were negative for CD31 and CD45 (Figure 2).

**Figure 3:**
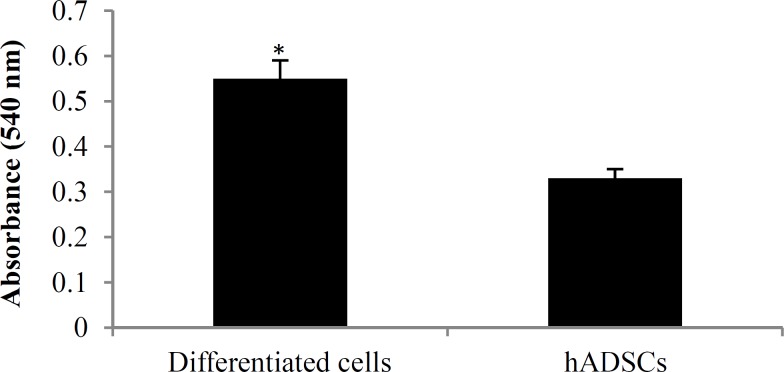
3-(4,5-dimethylthiazol-2-yl)-2,5-diphenyltetrazolium bromide (MTT) histogram of human adipose-derived stem cells (hADSCs) and differentiated cells

***Cell viability:*** Viability of hADSCs and differentiated cells was determined via MTT assay. The mean absorbance value was significantly increased in differentiated cells compared to hADSCs (P < 0.050) (Figure 3).

The mean absorbance value of the differentiated cells significantly increased as compared to hADSCs. ^*^P < 0.050

**Figure 4:**
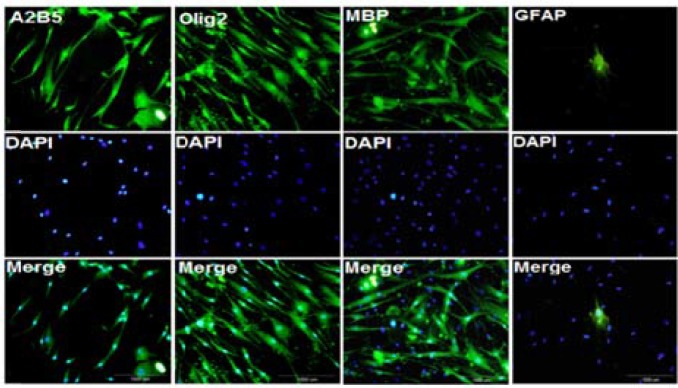
Immunocytochemistry of differentiated cells at the end of the cell differentiation

**Figure 5:**
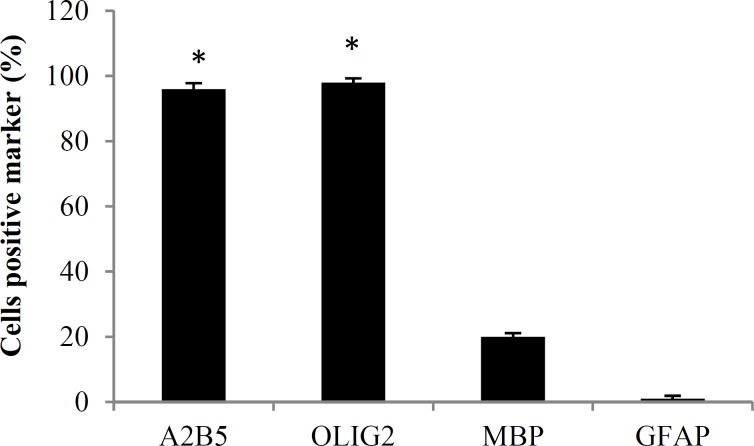
The mean percentage of cells positive marker at the end of the cell differentiation

***Immunocytochemistry study of differentiated cells:*** At the end of cell differentiation, immunocytochemistry technique was done for investigation of specific markers. Fluorescence microscopic images of separate samples showed that 96.0 ± 1.8 percent of differentiated cells expressed A2B5, and 98.0 ± 1.3 percent of them expressed Olig2, which are oligodendrocyte precursor markers. In addition, 20.0 ± 1.1 percent of differentiated cells expressed MBP which is mature oligodendrocyte marker (Figures 4 and 5), and a very low percent of them (1.0 ± 0.9 percent) expressed GFAP (astrocyte marker) which were significantly lower than Olig2 and A2B5 (P < 0.001).

The cell samples were stained separately with fluorescein isothiocyanate (FITC)-anti oligodendrocyte transcription factor (Olig2), FITC-anti A2B5, FITC-anti FITC- myelin basic protein (MBP), and FITC- anti glial fibrillary acidic protein (GFAP). 4',6-diamidino-2-phenylindole (DAPI) was used for nuclear counterstaining (blue) in order to show all the cells. High percentages of differentiated cells were expressed Olig2 and A2B5 (precursor oligodendrocyte markers) and a low percent of them were expressed MBP (mature oligodendrocyte marker) and GFAP (astrocyte cell marker) (Scale bar: 1000 µm).

The mean percentage of A2B5-, oligodendrocyte transcription factor (Olig2)-positive cells was signiﬁcantly increased compared to glial fibrillary acidic protein (GFAP)- and myelin basic protein (MBP)-positive cells.

^*^P < 0.001

**Figure 6:**
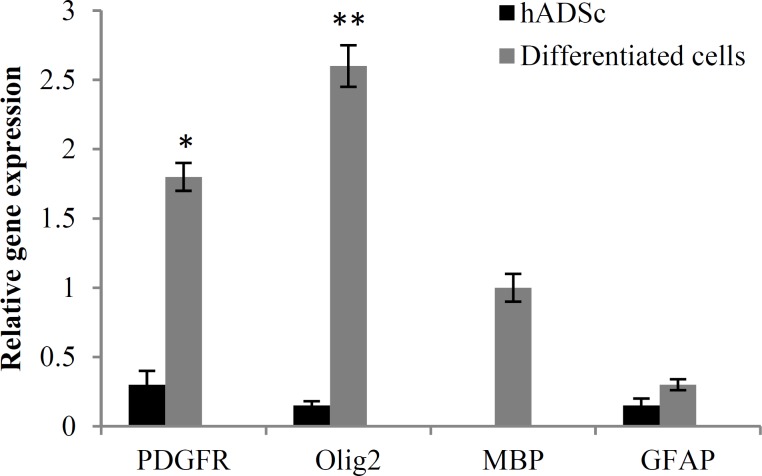
Comparative analysis of oligodendrocyte specific markers examined via real-time polymerase chain reaction (RT-PCR)

***RT-PCR results:*** After oligodendrocyte differentiation, total RNA extracted from differentiated cells in order to RT-PCR assay. To this end, GAPDH was used as a control marker. Our results revealed that the expression of Olig2 and PDGFRα markers were higher compare to MBP and GFAP markers which is consistent with our immunocytochemistry results. In addition, the mean expression level of Olig2 and PDGFRα genes increased significantly in differentiated cells in compared to control group (Figure 6).

Quantification was done in oligodendrocytes after normalization to glyceraldehyde-3-phosphate dehydrogenase (GAPDH). The mean expression level of oligodendrocyte transcription factor (Olig2) and PDGFRα genes increased significantly in differentiated cells in compared to control group.

^*^P < 0.010, ^**^P < 0.001

## Discussion

Stem cell therapy results are associated with many intrinsic and extrinsic risk factors. Since stem cells have several features resemble with cancer cells (such as long life span, apoptosis resistance, and pluri- or multipotency),^16^^,^^17^ these cells may be considered as potential candidates for malignant transformation. Therefore, the transplantation of fully differentiated cells instead of stem cells in order to diminish tumor genesis is a new idea. Although stem cells unlike fully differentiated cells may higher create cell-cell junction and communicate with host cells, in vitro differentiation of stem cells into other specific cell types will decrease the potency of these cells, and may reduce the risk of tumor organization.^18^

hADSCs which are a kind of adult stem cells have several specific features. Due to the abundance and availability, these cells may be one of the ideal sources for stem cell therapy.^19^ Previous studies have shown that hADSCs have the ability to differentiate into other cells of ectodermal, mesodermal, and endodermal lineage, and can promote cell differentiation and nerve protection via paracrine effects.^13^^,^^20^^,^^21^ In present study, for the first time, hADSCs were differentiated into oligodendrocyte cells in order to access a valuable cell source for later cell transplantation in MS disease. 

Some species of stem cells such as hematopoietic stem cells are relatively more numerous in men than in women.^22^ In addition, it has been reported that stem cells separated from the muscles of female mice are better at regenerating tissue than those taken from male mice.^23^ In similar experiment, Ogawa, et al. reported that adipose derived stem cells (ADSCs) which isolated from female mice have more ability to differentiate into adipocytes than those taken from male mice.^24^ Thus, it appears that the differentiation potential of ADSCs is closely related to sex differences. As a result, in this study, hADSCs were taken from healthy female donors, and then differentiated into oligodendrocyte progenitor cells.

A2B5 is a membrane epitope which typically express in oligodendrocyte precursor cells.^25^ Moreover, Olig2 is critical for oligodendrocyte and motor neuron differentiation.^26^ Thus, in this study, both Olig2 and A2B5 were assessed in differentiated cells. After immunocytochemistry, it was found that a high percentage of differentiated cells expressed special markers of oligodendrocyte precursor cells, and a low percentage of them expressed mature oligodendrocyte marker (MBP). In addition, a high expression of Olig2 and PDGFRα genes also was seen in differentiated cells which was higher than expression of MBP and GFAP genes. In similar experiments, other sources of stem cells such as spermatogonia stem cells, endometrial stromal cells, mouse-induced pluripotent stem cells, and human-induced pluripotent stem cells also differentiated into oligodendrocyte.^27^^-^^30^ The results of these studies revealed that 88-94 percent of differentiated cells expressed Olig2, and 75-92 percent of them expressed A2B5.

Therefore, the comparison of these results show that by hADSCs differentiation, we can achieve a higher percentage of precursor oligodendrocyte cells.

One hypothesis could be that hADSCs can secrete high levels of neurotrophic factors, which could support the cell differentiation.

In this study, the mean absorbance value of the differentiated cells significantly increased as compared to control group; which could be due to the presence of growth factors in cell differentiation medium.

Another relevant finding of our study is that a less than 3 percentage of differentiated cells were only positive for GFAP which shows the efficiency of in vitro differentiation of hADSCs into oligodendrocyte cells.

## Conclusion

Overall, the results of present study revealed that hADSCs are able to differentiate into oligodendrocyte precursor cells at a high level. Thus, due to the strong differentiation capacity of hADSCs and considering their ability to increase the neighboring cells viability through paracrine effects, it seems that hADSCs could be an ideal cell source for in vitro oligodendrocyte differentiation.

## Notes:

**How to cite this article:** Ghasemi N. Transdifferentiation of human adipose-derived mesenchymal stem cells into oligodendrocyte progenitor cells. Iran J Neurol 2018; 17(1): 24-30.
